# Evaluation of Faculty and Non-faculty Physicians’ Medication Errors in Outpatients’ Prescriptions in Shiraz, Iran

**Published:** 2018

**Authors:** Pegah Misagh, Afsaneh Vazin, Soha Namazi

**Affiliations:** a *Department of Pharmacotherapy, School of Pharmacy, Shiraz University of Medical Sciences, Shiraz, Iran. *; b *Department of Pharmacotherapy, School of Pharmacy, Shiraz University of Medical Sciences, Shiraz, Iran. *; c *Department of Pharmacotherapy, School of Pharmacy, Tehran University of Medical Sciences, Tehran , Iran.*

**Keywords:** Medication error, Prescription error, Outpatient, Prescription, Faculty physicians, Non-faculty physicians

## Abstract

This study was aimed at finding the occurrence rate of prescription errors in the outpatients› prescriptions written by faculty and non-faculty physicians practicing in Shiraz, Iran. In this cross-sectional study 2000 outpatient prescriptions were randomly collected from pharmacies affiliated with Shiraz University of Medical Sciences (SUMS) and social security insurance in Shiraz, Iran. Patient information including age, weight, diagnosis and chief complain were recorded. Physicians ‘characteristics were extracted from prescriptions. Prescription errors including errors in spelling, instruction, strength, dosage form and quantity as well as drug-drug interactions and contraindications were identified. The mean ± SD age of patients was 37.91 ± 21.10 years. Most of the patients were male (77.15%) and 81.50% of patients were adults. The average total number of drugs per prescription was 3.19 ± 1.60. The mean ± SD of prescription errors was 7.38 ± 4.06. Spelling error (26.4%), instruction error (21.03%), and strength error (19.18%) were the most frequent prescription errors. The mean ± SD of prescription errors was 7.83 ± 4.2 and 6.93 ± 3.88 in non-faculty and faculty physicians, respectively (P < 0.05). Number of prescription errors increased significantly as the number of prescribed drugs increased. All prescriptions had at least one error. The rate of prescription errors was higher in non-faculty physicians. Number of prescription errors related with the prescribed drugs in the prescription.

## Introduction

 “A prescription drug order is a lawful written instruction from a licensed physician or other medical practitioner to a licensed pharmacist regarding the compounding or dispensing and administration of drug/s or other medical services to the patient” (1). Prescriptions are intended to improve patients’ quality of life by curing a disease, reducing or eliminating the symptoms, slowing or arresting disease process, or preventing a disease or its symptoms from appearing in the first place (2). Rational prescribing is based on a sound knowledge as it requires understanding the pathophysiology of the diseases as well as clinical pharmacology of the drugs used (3). The purpose is to achieve maximum benefits while respecting the patient’s choice and minimizing costs and risks (4). Irrational drug prescribing has been identified as one of the risk factors for medication error (ME) (5). A ME is defined as “a failure in the treatment process that leads to, or has the potential to lead to, or harm the patient.” (6).Medication Errors (MEs) compromise patient confidence in the healthcare system and can increase healthcare costs (7). According to the reports of the Institute of Medicine 44,000 to 98,000 Americans die each year due to (MEs) (8) and the annual cost is 77 billion dollars (9). It has been predicted that the rate of serious MEs in the USA is approximately 7% (10). The frequency of (MEs) was reported and analyzed in different parts of healthy stages in Iran (11-13).

 Potentially errors can occur at any steps in the medication process including prescribing, transcribing, dispensing, administering and monitoring (14). The prescribing stage is mostly prone to errors, accounting for 49% of MEs (14). Prescription error is defined as “any preventable event that may cause or lead to inappropriate medication or patient harm when the medication is in the control of the health care professional, patient or consumer” (15). Several causes have been reported for occurrence of prescription errors, such as lack of knowledge and illegible physicians' handwritings (16). The components of a prescription should be clearly written, free of omission and commission errors (1, 16). Identifying of medication errors in outpatient prescriptions is difficult and there are few data available about the frequency and impact of these errors (17). One study has shown that 15-21% of outpatient prescriptions contain at least one error (17). In another study by Buurma *et al*. (18) 16% of patients reported a ME and two-thirds of cases were in outpatient settings. One study (19) has reported that MEs can occur at any age however it has a higher rate of occurrence in pediatrics and elderly patients compared to adults.

 The aim of this study is to identify common types of handwritten MEs in outpatient prescriptions and compare the rate of MEs between physicians who are faculty members affiliated to Shiraz University of Medical Sciences (SUMS) and those physicians practicing in Shiraz who are not affiliated to the university (non-faculty).

## Experimental

*Data collection*

This study was a cross-sectional survey of 2000 outpatient prescriptions received at educational pharmacies affiliated with SUMS and health insurance companies in Shiraz, Iran. Shiraz is the capital of Fars province in Iran. These prescriptions were selected randomly from March to September 2012, regardless of sex, race and age. Ethics committee of SUMS approved this study and information related to patients and physicians remained confidential.

Prescriptions were categorized into two groups: faculty and non-faculty according to the physicians’ stamps. Prescriptions were analyzed based on gender of physician and patient, age of patient, academic position in case of faculty physicians (assistant professor , associate professor, or full professor), physicianꞌs level of training (general practitioner, specialist or fellowship) , field of practice and number of generic and brand drugs per prescription.

The commission and omission errors were determined in this study as: number of messy and illegible spelling of drugꞌs name, number of dosage form errors, strength errors, instruction errors (dosage, route, frequency and duration), quantity errors, drug-drug interactions, contraindications and combination errors. We also determined if the chief complain and diagnosis was mentioned in the footnote of prescription or not. According to the regulation of prescribing in Iran. Physicians should write the chief complain and diagnosis on the prescription. In the current article commission and omission errors are presented in a single output. In this investigation “Instruction errors” indicates the wrong dosage regimen prescribed for a specific disease. In order to calculate the approximate range of dosages for pediatrics, the weight-age Table was used because Iranian physicians usually do not include weight of patients on prescriptions. If the prescribed dosage did not match the standard dosage, it was considered as erroneous. Contraindications based on appropriateness of a drug for a specific age or sex, as there was the only information available. Combination errors were determined using the prescribed drugꞌs strength, dosage form, quantity, and instruction regimen for the specific disease in each prescription by protocols defined in references (20, 21). In addition, prescribing drugs in the same pharmacology class (duplicate drugs) were considered as combination errors.

Spelling, dosage form and strength were determined by drug information handbook of Lexi® (22) and Iranpharma (the pharmacopeia of Iran) (23). Other prescription errors were identified based on Medscape® (24) database software and drug information handbook of Lexi® (22) and Lexi-Comp drug-drug interactions software version 1.9.1 (25) alongside clinical judgment of clinical pharmacist.

An experienced pharmacist reviewed prescriptions to identify and categorize likely prescription errors. All prescriptions with suspected prescription errors were double checked and evaluated by two clinical pharmacists affiliated to SUMS to confirm the errors.

*Statistical Analysis*

Data analysis was performed using Statistical Package for Social Sciences (SPSS version 20 “IBM© SPSS Statistic) software. Continuous data is presented as mean ± SD and categorical data is shown as frequency or percent. The relationships between number of drugs per prescription, patient and physician characteristics and rate of occurrence of prescription errors were examined. The differences between the mean ± SD of two continuous data groups were examined with t-test. ANOVA was used to determine the difference between means of more than two continuous variable groups. Post-hoc analysis was examined where ANOVA-test was significant. Chi-square test was processed to determine the difference between categorical data. *P*-value less than 0.05 were considered as significant difference.

For further evaluation prescriptions were categorized into 3 groups based on the number of prescribed drugs: 1-2, 3-4, and ≥ 5 drugs in each prescription. In each group, the ratio of total errors per number of prescribed drugs was calculated.

## Results

Total of 2000 prescriptions were collected randomly during six months.1000 prescriptions were written by faculty members and 1000 prescriptions by non-faculty. Table 1 shows the characteristics of physicians.

By studying the field of specialty of physicians, it was found that the majority (9.50%) of physicians were neurologists and minority (0.30%) were anesthesiologists. In addition, the least number of faculty members were anesthesiologists while for non-faculty members infectious disease specialists were the minority group. Most female physicians were gastroenterologists while most male physicians were neurologists.

In this study only 1 out of 2000 prescriptions contained chief complain and 21 of them contained diagnosis (faculty member, 1.8% vs. non-faculty, 0.30%, *p* > 0.05).

The mean ± SD age of patients was 37.91 ± 21.10 years. The differences between age of males and females were not significant (*P*-value = 0.26). Most of the patients (*N* = 1509, 81.5 %) were adults. The mean ± SD prescription errors was 7.38 ± 4.06 (*N* = 14764) (excluding chief complain and diagnosis). The minimum and maximum errors observed in one prescription were 1 and 26 errors, respectively. Femalesꞌ prescription had significantly higher errors than malesꞌ prescriptions (7.63 ± 4.17 vs. 7.04 ± 3.90, *P* = 0.00).

*Prescription errors are shown in* Table 2

The highest rate of prescription errors (49.02%) were observed in psychiatrists. The rate of occurrence of prescription errors were higher in adult patients than pediatrics; however this difference was not significant (7.46 ± 4.07 vs. 7.07 ± 4.11, *P*-value = 0.11). One thousand and ninety drugs were prescribed by trade names. The mean ± SE of trade names were (0.55 ± 0.017). Faculty physicians used trade name less frequently than non-faculty members, significantly (0.48 ± 0.021 vs. 0.61 ± 0.027, *P* = 0.00).

As mentioned in the statistical analysis, the ratio of errors to total number of drugs in each prescription was calculated. Errors are shown in the Table 3. The difference between prescription errors based on total number of drugs prescribed was significant**. **The mean of errors in all groups of prescription errors increased significantly as number of prescribed drugs increased.

In the faculty member group academic grade did not have a significant effect on rate of occurrence of ME (*P* > 0.05).

## Discussion

Our study has demonstrated a wide range of prescription errors associated with 2000 prescriptions from faculty and non-faculty physicians in Shiraz, Iran. We documented 14764 errors from 2000 prescriptions. All prescriptions (100%) had at least one error. The maximum prescription error was 26 errors in one prescription. The total MEs in our study were greater than previous studies in Iran. In a study by Vazin *et al.* 68.5% of prescription had an error (12), Abbasinazari *et al*. (11) detected 262 errors in 132 patients (1.98 per each patients) and in Malaysia Kuan Mon Ni *et al*. (26) only 13 out of the 397 prescriptions screened complied with all the legal requirements. T. Khoja *et al*. (27) found that 18.7% of their prescriptions (5299) contained an error. Perwitasari *et al*. (28) reported 98.69% error in their study which was similar to our result. This indicates a need for the physicians to further emphasize the necessity of writing prescriptions clearly, and completely. Several causes of the high rate of prescription errors in our study compared to other studies were handwritten prescriptions, difference in references used for identify prescription errors and different populations studied.

The mean ± SD errors in prescriptions written by faculty members was significantly lower than the non-faculty members (6.93 ± 3.88 Vs 7.83 ± 4.2, *P*-value < 0.001). Faculty members practice in the academic area and have more complicated patients daily. They have the responsibility to educate the medical students and therefore need to keep themselves up to date.

The mean number of drugs prescribed per prescription in our study was 3.21 ± 1.71. This was similar to previous reports (3.0-4.5) from Nigeria (29, 30) and higher than a study in Urmia (31). (*N* = 2.54 ± 0.58; 2.26 ± 0.43 for faculty vs. 2.65 ± 0.59 for non-faculty, *P*-value < 0.001). This finding may be related to the greater awareness of faculty members about the existence of Rational Use of Drugs (RUD) committee in the university as an official authority for evaluation of physicians (31). Based on the latest available annual repoort of Iranian RUD committee in 2011, the mean number of drugs per prescription for all prescriber in Iran was 3.05 (31). Developing countries with programs promoting rational drug use as well as standards have described lower drug prescription errors rates, such as primary health care center in Jordan (2.3%), primary health care center in Tanzania (2.9%) and private hospitals in Uzbekistan (2.9%) (32, 33). Poly pharmacy has been reported as one of the causes of prescription errors (such as increasing drug-drug interactions and combination error), increasing costs for patients and confusing patients (34).

In our study average patientsꞌ age was 37.91 ± 21.10 years. About 7.45% of our prescriptions didnꞌt have any information about age or date of birth. In our study, we had a lower rate of lack of patient’s age data compared to a research conducted at Government Hospitalian Yogyakarta Indonesia in 2010 (52.4% of 229) registered outpatients (28). One study in Oman reported that patientꞌs age is one of the most common omission errors and itꞌs frequency of occurrence was more than 72% (35). The age has a great impact on the prescription and it is a very important variable in terms of dose and dosage form. The omission of age in prescriptions could be a reflection of the fact that some physicians does not appreciate the legal status of a prescription order (28).

The mean ± SD spelling error was 1.95 ± 0.03 (11% in 2000 prescriptions) in our study which is higher than research done by Kuan Mun Ni *et al*. (26) who screened the prescriptions and were unable to read about 7% of prescriptions in their sample. In this study, 6.11% in 229 prescriptions contained spelling errors.

In our study, the mean ± SD strength error was 1.42 ± 0.03 (9.20% in 2000 prescriptions) and dosage form errors were 0.68 ± 0.02 (4.85% in 2000 prescriptions). Kuan Mon Ni *et al*ꞌs study (26) 56.26% (485/862) of drugs were prescribed without strength specifications. Our study indicated lower errors than the mentioned study in Indonesia. Kuan Mon Ni *et al*. (26) reported that among 391 studied prescriptions, 27out of 862 drugs had wrong written dosage forms and 314 out of 862 drugs didn’t have dosage forms.

**Table 1:** The characteristics of study physicians (N = 2000).

**Characteristics of physicians**		**Faculty prescription** **(N = 1000)**	**Non-faculty prescription** **(N = 1000)**	**Total prescription** **(N = 2000)**
Sex	Male	840 (84%)	703 (70.3%)	1543 (77.15%)
	Female	160 (16%)	297 (29.7%)	457 (22.85%)
Field of Practice	General-physician	-	292 (29.2%)	292 (14.6%)
	Specialist	446 (44.6%)	534 (53.4%)	980 (49%)
	Fellowship	554 (55.4%)	174 (17.4%)	728 (36.4%)
Academicals Grade	Associate professor	461 (46.1%)	-	461 (23.05%)
	Assistant professor	360 (36%)	-	360 (18%)
	Full-professor	179 (17.9%)	-	179 (8.95%)

**Table 2.** Total number of errors extracted from outpatients' prescriptions (Number of prescription = 2000).

**Errors**	**Faculty prescriptions** **(N = 1000)**	**Non-faculty prescriptions** **(N = 1000)**	**Total prescriptions (N = 2000)**	***P*** **-value**
Total numbers of prescribed drugs mean ± SD(N)	3.02 ± 1.72 (3023)	3.39 ± 1.69 (3388)	3.21 ± 1.71 (6411)	0.00
Spelling error mean ± SD(N)	1.66 ± 0.04 (1661)	2.24 ± 0.04 (2238)	1.95 ± 0.03 (3899)	0.00
Instruction error mean ± SD(N)	1.44 ± 0.04 (1444)	1.66 ± 0.03 (1662)	1.55 ± 0.03 (3106)	0.00
Strength error mean ± SD(N)	1.21 ± 0.03 (1208)	1.62 ± 0.045 (1624)	1.42 ± 0.03 (2832)	0.00
Dosage error mean ± SD(N)	0.80 ± 0.04 (799)	0.56 ± 0.031 (563)	0.68 ± 0.02 (1362)	0.00
Quantity error mean ± SD(N)	0.47 ± 0.02 (472)	0.43 ± 0.026 (429)	0.45 ± 0.01 (901)	0.22
Combination error Mean ± SD(N)	0.21 ± 0.01 (208)	0.22 ± 0.01 (225)	0.22 ± 0.00 (433)	0.356
Contraindication mean ± SD(N)	0.03 ± 0.00 (34)	0.02 ± 0.00 (18)	0.03 ± 0.00 (52)	0.02
Drug-drug interaction mean ± SD(N)	0.12 ± 0.01 (124)	0.08 ± 0.01 (76)	0.1 ± 0.00 (200)	0.00
Total prescription errors mean ± SD(N)	6.93 ± 3.88 (6232)	7.83 ± 4.2 (7832)	7.38 ± 4.06 (14764)	0.00

**Table 3:** Total prescription errors based on total numbers of drugs prescribed.

**Total numbers of drugs per prescription**	**The Mean ± SD prescription errors based on total numbers of drugs per prescription**					
	**Spelling error**	**Dosage form error**	**Strength error**	**Instruction error**	**Quantity error**	**Total errors**
1-2	1.39 ± 0.49	0.39 ± 0.02	0.72 ± 0.03	0.86 ± 0.03	0.22 ± 0.01	2444
3-4	2.35 ± 0.93	0.67 ± 0.03	1.70 ± 0.04	1.65 ± 0.03	0.52 ± 0.02	6202
≥5	3.66 ± 1.56	1.41 ± 0.11	2.4 ± 0.09	2.91 ± 0.10	0.75 ± 0.06	4139
*P*-value	0.00	0.00	0.00	0.00	0.00	-

Amount of omission and commission errors were 39.55 % (26). If the strength of a drug is incorrect, it may lead to more serious consequences compared to when the strength is not written at all. If the drug is only available in a fixed dosage form, this type of error could be easily identified and rectified. An incorrect dosage form does not lead to serious consequences unless the strength or the frequency of use of that dosage form is also incorrect (26).

In our survey, the mean ± SD instruction error was 1.55 ± 0.03 (10.64% in 2000 prescriptions), which is lower than the results of previous studies by Nadiya *et al.* (35) and Perwitasari *et al.* (28) who reported the instruction errors to be 46.33% and 26.43%, respectively. Based on a survey conducted in a general practice in USA, the highest frequency of errors was due to incomplete or missing instruction (36). Instructions should be clear, complete and should provide careful instructions for pharmacists about the patient’s situation (35).

Our results showed that pediatric patients had 2421 (17.69%) errors in 2000 prescriptions. Pediatrics pose a unique set of risks of prescription errors, predominantly because of the need to make dosage calculations, which are individually based on patientꞌs weight. Therefore, lack of patientꞌs weight, may result in overdose or under dose and patients would not benefit from treatment (37, 38). Lack of appropriate information such as patientꞌs weight makes it difficult for pharmacists to carry out a prescription (37). In our study, none of the prescriptions stated patient’s weight. It is possible that the physicians consider the patientꞌs weight (especially pediatrics) before prescribing and calculate the dose, therefore this may not lead to an error. In the study by Perwitasari *et al*., Only 3 prescriptions included patient’s weight and 98.69% of the prescriptions lacked this variable (28). 

The mean ± SD quantity errors were 0.45 ± 0.01 (4.17% in 2000 prescriptions). In a study by Kuan Mun Ni *et al*. (26), they reported that 50 out of 862 (5.8%) drugs had quantity errors. Prescriptions should state the quantity of each drug. Although some drugs may be given on “as required” basis, the physician is still the best judge on the total quantity to be supplied based on the patient medical requirement. Even for dermatological, eye, ear, and nasal preparations, documentation of the amount to be supplied is still necessary (26). In this study, quantity was considered as an indicator for treatment duration. If the quantity of drug/drugs is not enough the duration of treatment would not be completed which can increase the probable risk of treatment failure. On the other hand, excess amount of drugs may cause adverse effects, increase patient’s cost and drug waste (34).

Combination therapy is considered to increase the efficacy of drug therapy. However, if co-prescribed drugs have the same mechanism of action or they antagonize each other, a combination error is observed. In our study, 433 (2.93%) in 2000 prescriptions had combination errors. We could not find any published study about these types of errors.

In the present study 52 (2.60%) in 2000 prescriptions had contraindication error. Our rate was higher than the reported rates by Teixeira *et al*. in Brazil (0.4% of the prescriptions contained contraindications) (39) and Guedon *et al*. in France (0.4% contraindication in outpatient prescriptions) (40). Describing “chief complain” and “diagnosis” help us identify contraindication but our physicians did not include these data. Thus we reported contraindication error only based on sex and age of patients. Therefore, our findings may be underestimated.

In this study 1090 (40.7%) drugs were prescribed by trade names. The rate of drugs prescribed by trade names was lower than rate of using generic names (7-7.3%) in other studies (30, 33). Generic prescribing makes is possible to dispense various brands of drugs that are cheaper than or are as effective as proprietary brands (4). The several suggestions for this type of prescription error in our study are as follows: 1-physicians are not certain enough about quality of generic drugs. 2-Trade names are used because physicians are more familiar with trade names than generic names. 3-when some drugs are launched in Iranꞌs market for the first time, they often are introduced by their trade names and prescribing drugs using trade name has become a routine.

Overall, the rate of occurrence of prescription errors in this study was higher than others (26-28). Some reasons for this difference may be: 1) Differences in prescribing systems between our country and other countries; 2) Use of different references to identify errors, 3) differences in sample size and design of studies such as different ways of selecting prescriptions.

Our study had some limitations: 1) Prescriptions were limited to handwritten prescriptions. Verbal instructions of physicians to patients remain unknown. 2) This study covered Shiraz city. Since certain types of drugs are available in a specific city, it’s possible that our study is restricted to certain types of drugs. Our results canꞌt be generalized to another university of Iran. For example in some therapeutic centers in Iran, the prescription control committee can be very strict making physicians to write prescriptions more carefully. 3) Our study’s focus was mainly on prescription errors and was not designed to detect adverse drug events or other complications of medication errors. 4) Vast majority of physicians did not state chief complain and diagnosis on their prescriptions, therefore it was not possible to make accurate judgments on all prescriptions especially regarding combination and contraindication errors. 5) One of the reasons for diversity in our results may be due to the fact that various types of physicians were included in our study. Prescriptions were selected using random sampling and there was no criteria or limits on the number of physicians in this study. 6) Because our study was retrospective, some contraindication errors, combination errors and quantity errors may not have a true error.
